# Predicting Fall-Related Deaths in Japan by Seasonality

**DOI:** 10.7759/cureus.107444

**Published:** 2026-04-21

**Authors:** Takashi Miyazawa, Nobuhiro Nasu, Takehiko Asaga, Hiromi Suzuki, Nobuyuki Miyatake

**Affiliations:** 1 Department of Hygiene, Faculty of Medicine, Kagawa University, Kagawa, JPN; 2 Department of Orthopedic Surgery, Ina City National Health Insurance Osafuji Clinic, Nagano, JPN; 3 Department of Physical Medicine and Rehabilitation, Okayama Healthcare Professional University, Okayama, JPN; 4 Department of Intensive Care Medicine, Faculty of Medicine, Kagawa University, Kagawa, JPN

**Keywords:** air temperature parameters, falls, prediction, prophet analysis, seasonality

## Abstract

Objective: In Japan, fall-related deaths have been increasing in parallel with rapid population aging, yet nationwide studies remain scarce. Against this background, the establishment of prevention strategies for falls has become a public health challenge in Japan. We herein attempted to predict fall-related deaths in consideration of seasonality.

Methods: In this ecological study, we collected data on the number of fall-related deaths (per month and per 100,000 people) in Japan between 2017 and 2023 and the number of fall-related deaths (per year and per 100,000 people) in each of the 47 prefectures between 2021 and 2023 from the official website of the Ministry of Internal Affairs and Communications. We also used air temperature parameters from the official website of the Japan Meteorological Agency and the aging rate from the official website of the Cabinet Office, Japan.

Results and conclusion: The number of fall-related deaths was the highest in the month of December and was also significantly higher in December than in July (p = 0.0194) and August (p = 0.0192). An analysis of data from each of the 47 prefectures showed that the number of fall-related deaths correlated with air parameters. A Prophet (Meta, Menlo Park, CA, USA) analysis was performed to predict the number of fall-related deaths in Japan for the next three years and revealed an increase with seasonal patterns. The number of fall-related deaths is predicted to increase with seasonality in Japan.

## Introduction

Fractures resulting from falls may result in a bedridden status, which reduces quality of life (QoL) and may lead to death. Therefore, the establishment of proper prevention and management strategies for falls is urgently required in Japan. According to Cabinet Office surveys in Japan, 10.6% (2005) [1] and 9.5% (2010) [2] of adults aged 60 years and older had at least one fall inside their home in the previous year. In 2005, one-year fall rates inside the home were 6.8% at 60 to 64 years, 8.5% at 65 to 69 years, 10.1% at 70 to 74 years, 11.3% at 75 to 79 years, 18.6% at 80 to 84 years, and 25.3% at 85 years and older, indicating an age-related increase in the risk of falls [1]. Furthermore, among adults aged 60 years and older who fell at home, 62.5% in 2005 [1] and 66.7% in 2010 were injured [2]. Since 2010, the number of deaths due to falls has exceeded those due to traffic accidents [3] and was 3.4-fold higher than the number of traffic accident deaths in 2024 [4]. Individuals requiring long-term care due to falls and fractures also ranked third (13.9%) among those in long-term care due to all causes in 2022 [5]. Falls and fractures impose a heavy caregiving burden, causing stress in 70% of caregivers and weekly labor losses of 43,000 yen [6]. As the population ages, the number of individuals requiring support or care has been steadily increasing, and the societal burden associated with falls and fractures is growing.

Meteorological parameters, particularly air temperature parameters, have been closely associated with a number of health outcomes, i.e., cerebrovascular disease [7], pneumonia [8], and heart disease [9]. We also reported a relationship between air temperature parameters and health outcomes, i.e., the total number of deaths [10], the number of ambulance transports due to heat stroke [11], and the number of deaths due to renal failure [12]. In addition, Uehara et al. demonstrated that the number of deaths due to falls was associated with air temperature parameters in the 23 wards of Tokyo, Japan [13].

A time series analysis is a method for forecasting the future, and techniques include moving averages, autoregressive integrated moving average (ARIMA), and long short-term memory (LSTM) [14]. The Prophet (Meta, Menlo Park, CA, USA) analysis, originally developed by Facebook, is easy to operate, suitable for forecasting future data with seasonality and periodicity, and offers the advantage of easily visualizing results, particularly in epidemiological fields [15]. Previous studies predicted the number of diseases using a Prophet analysis [16-19].

However, predictions of the number of fall-related deaths in Japan have yet to be conducted using a time series analysis, particularly a Prophet analysis.Therefore, to obtain data for preventing future fall-related deaths, we aimed to predict fall-related deaths in Japan for the next three years using a Prophet time-series analysis that accounts for seasonality as the primary objective and to examine the relationships between fall-related deaths and air temperature indices and the aging rate as the secondary objective.

## Materials and methods

Data collection

We obtained monthly data on the number of fall-related deaths (per 100,000 people) in Japan between 2017 and 2023 from the official website of the Ministry of Internal Affairs and Communications [20]. Annual data on the number of fall-related deaths (per 100,000 people) in each of the 47 prefectures between 2021 and 2023 were also collected from the official website of the Vital Statistics of Japan, accessed via the e-Stat portal [21]. We used annual air temperature parameters, i.e., mean air temperature (℃), the highest air temperature (℃), the lowest air temperature (℃), the mean of the highest air temperature (℃), and the mean of the lowest air temperature (℃) between 2021 and 2023 in each of the 47 prefectures in Japan from the official website of the Japan Meteorological Agency [22]. Annual data on aging rates from 2021 [23], 2022 [24], and 2023 [25] in each of the 47 prefectures in Japan were acquired from the official website of the Cabinet Office, Japan.

Data analysis

Data were expressed as mean ± standard deviation (SD). (Table 1). The number of fall-related deaths each month in Japan was compared using the Kruskal-Wallis test and Steel’s test [26]. A simple correlation analysis was used to evaluate the relationship between the number of fall-related deaths and air temperature parameters, and a multiple regression analysis was performed to adjust for the aging rate in 47 prefectures in Japan. We converted the 'year/month' variable into a date format (first day of each month) and ordered the data chronologically. The outcome was the monthly fall-related death rate per 100,000 people, and we confirmed that there were no missing values, so imputation was not required. Based on monthly data from 2017 to 2023, we predicted the number of fall-related deaths using a Prophet analysis, whose accuracy has been demonstrated [16-19]. Time-series forecasting was performed using the Prophet package in R (The R Project for Statistical Computing, Vienna, AUT).

In the Prophet settings, we enabled yearly seasonality and disabled weekly and daily seasonality. We specified a 36-month forecasting horizon with a 95% prediction interval and used the default setting for the trend and changepoints. Statistical analyses were conducted using JMP Pro 18 (JMP Statistical Discovery LLC, Cary, NC, USA) and R, which are free and open-source software programs. We did not calculate performance metrics such as mean absolute error (MAE) or root mean square error (RMSE) in this study. To support reproducibility, the R code used in the analysis is available from the corresponding author upon reasonable request.

Ethics approval

All data used in this investigation and analysis were publicly available data from Japanese government agencies. No personal data was used. Based on Japanese medical research guidelines, approval from an ethics committee was not required for this study.

## Results

**Figure 1:**
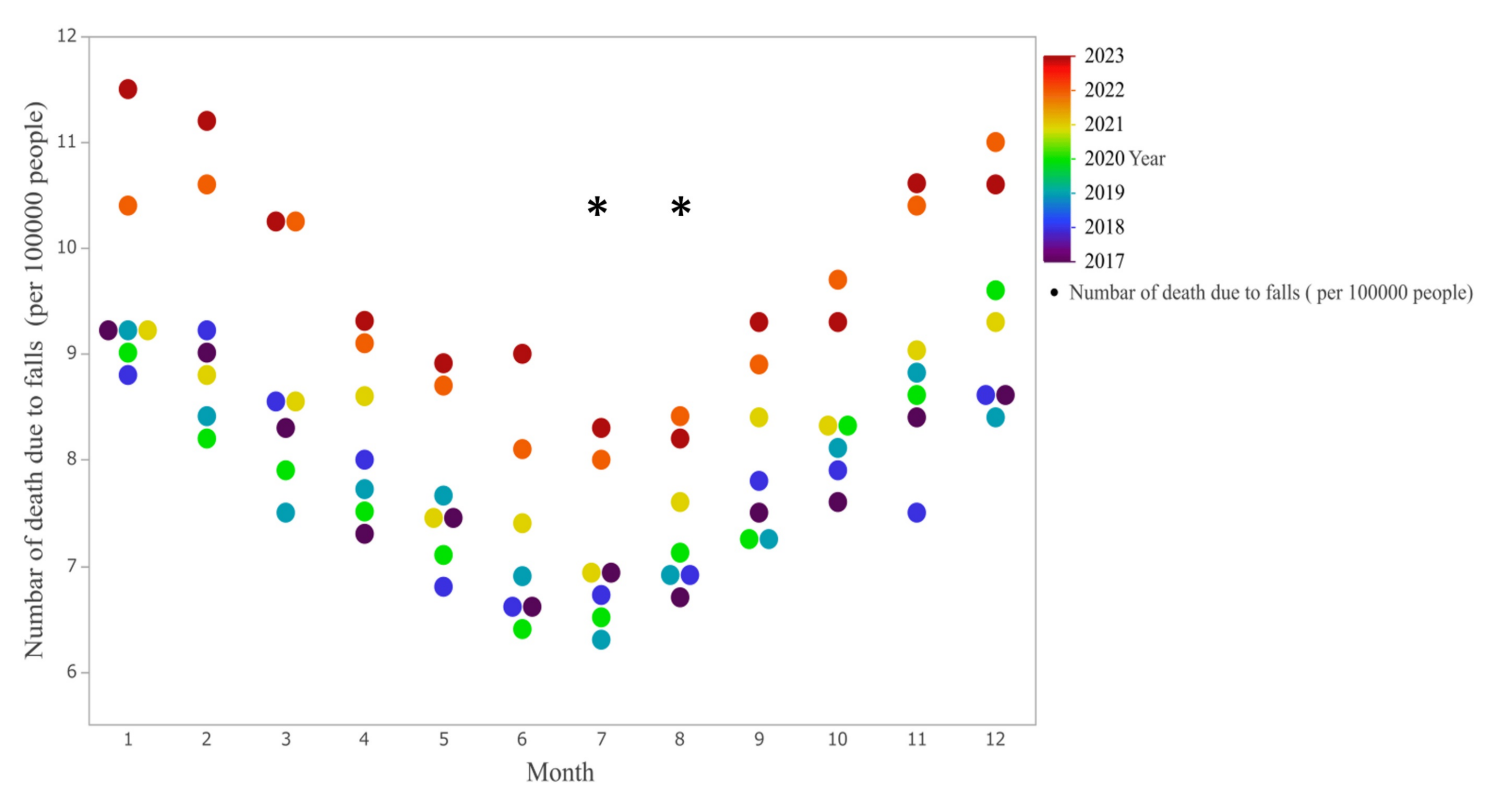
Comparison of number of deaths due to falls per 100,000 people classified by months *Significant difference from December

Figure 1 shows a comparison of the number of fall-related deaths in each month in Japan between 2017 and 2023. The number of fall-related deaths was the highest in December (9.4/100,000) and was also significantly higher in December than in July (p = 0.0194) and August (p = 0.0192).

The number of fall-related deaths and air temperature parameters are shown in Table 1. The number of fall-related deaths (per 100,000) in the 47 prefectures of Japan in the years 2021, 2022, and 2023 was 9.3±1.8, 10.6±2.2, and 10.8±2.1, respectively.

**Table 1:** Number of deaths due to falls and air temperature parameters in 47 prefectures of Japan

Variable	Mean	SD	Minimum	Maximum
Number of deaths due to falls (per 100,000)	10.2	±2.1	4.2	16.5
Elderly population ratio (%)	31.3	±3.2	22.7	38.8
Mean air temperature (℃)	16.3	±2.2	10.2	23.8
Highest air temperature (℃)	37	±1.3	32.9	39.5
Lowest air temperature (℃)	-4.1	±3.5	-13.2	11.7
Mean of the highest air temperature (℃)	21	±2.1	14.1	26.6
Mean of the lowest air temperature (℃)	12.3	±2.4	6.5	21.6

We then examined the relationship between the number of fall-related deaths (per 100,000) and air temperature parameters in the years 2021, 2022, and 2023. In 2021, the number of fall-related deaths correlated with the lowest air temperature. In 2022, the number of fall-related deaths correlated with the mean air temperature and the lowest air temperature. In 2023, the number of fall-related deaths correlated with some air temperature parameters, such as the mean air temperature, the lowest air temperature, the mean of the highest air temperature, and the mean of the lowest air temperature (Table 2). However, the relationship between the number of fall-related deaths and these air temperature parameters weakened after adjustments for aging rates in the 47 prefectures of Japan (Table 3).

**Table 2:** Simple correlation analysis between number of deaths due to falls and air temperature parameters in 47 prefectures of Japan Numbers highlighted in bold are p < 0.05

Parameter	2021		2022		2023	
	r	p	r	p	r	p
Mean air temperature (℃)	-0.215	0.146	-0.289	0.048	-0.409	0.004
Highest air temperature (℃)	0.17	0.253	-0.092	0.538	0.155	0.295
Lowest air temperature (℃)	-0.321	0.027	-0.385	0.007	-0.442	0.001
Mean of the highest air temperature (℃)	-0.147	0.322	-0.212	0.152	-0.3	0.04
Mean of the lowest air temperature (℃)	-0.266	0.069	-0.334	0.021	-0.453	0.001

**Table 3:** Multiple regression analysis between number of deaths due to falls and air temperature parameters by adjusting elderly population ratio in 47 prefectures of Japan VIF: Variance inflation factor

Variables	2021			2022			2023		
	β	p	VIF	β	p	VIF	β	p	VIF
Mean air temperature (℃)	0.098	0.313	1.162	-0.018	0.871	1.164	-0.129	0.29	1.248
Highest air temperature (℃)	0.148	0.097	1.001	0.113	0.29	1.078	0.196	0.07	1.003
Lowest air temperature (℃)	0.069	0.494	1.281	-0.098	0.371	1.144	-0.175	0.15	1.241
Mean of the highest air temperature (℃)	0.138	0.146	1.128	0.032	0.769	1.133	-0.002	0.981	1.234
Mean of the lowest air temperature (℃)	0.065	0.507	1.192	-0.052	0.644	1.187	-0.189	0.118	1.24

**Figure 2:**
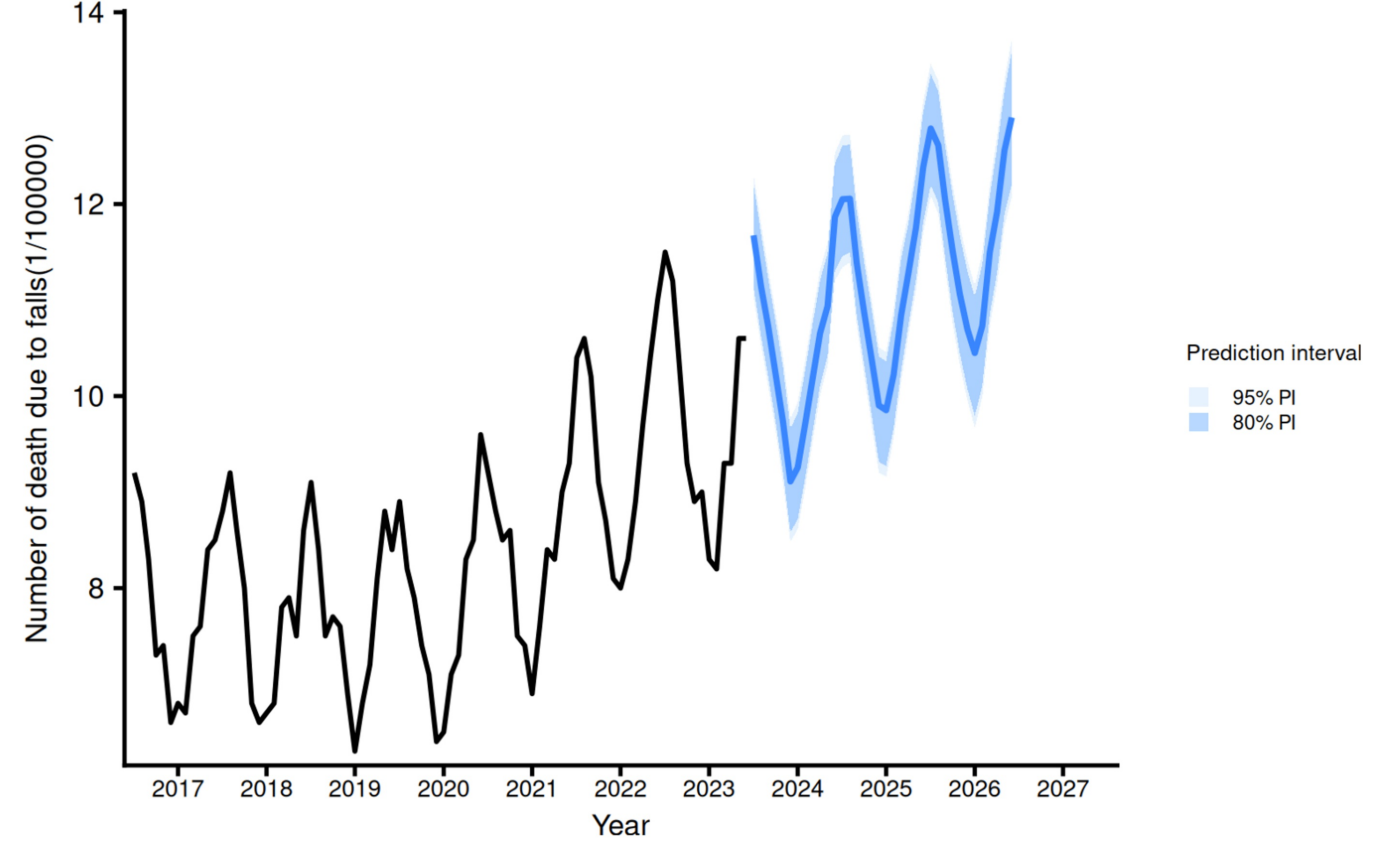
Prediction of number of deaths due to falls (per 100,000 people) up to the year 2027 using the Prophet (by Meta) analysis

We predicted the number of fall-related deaths for the next three years using the Prophet model of monthly data from 2017 to 2023 (Figure 2). The results obtained showed an increase in the number of fall-related deaths with seasonality.

## Discussion

We attempted to predict fall-related deaths in Japan using the Prophet analysis, and the results obtained revealed an increase with seasonality. The Prophet is a library developed by Meta (formerly Facebook, now Meta) for time series analysis. It is available for both Python (Python Software Foundation, Wilmington, DE, USA) and R, and its key feature is the ability to enable easy trend extraction and forecasting without specialized knowledge of time series analysis. Zrieq et al. analyzed historical COVID-19 data from March 2, 2020, to June 20, 2022, to forecast the trajectory of the pandemic [16]. Bera et al. performed a time series analysis with the Prophet model to examine 15 cities in Uttar Pradesh, India, and showed the extent to which the government’s clean air program reduced particulate matter (PM)10 levels compared with the levels that would have been expected in the absence of the program [17]. Ryu et al. investigated changes in hospital utilization by patients with schizophrenia during the last 10 years in Korea and demonstrated that changes in hospital use were accurately predicted [18]. In Japan, Arai predicted suicide rates following the 2024 Noto Peninsula earthquake [19]. Collectively, these findings indicate the utility of the Prophet analysis for forecasting. In the present study, we predicted the number of fall-related deaths in Japan using the Prophet analysis, and the results obtained show an increase in the number of fall-related deaths in the future. Thus, strategies need to be established to prevent fall-related deaths in Japan.

Falls mainly occur during the winter months, demonstrating a seasonal pattern. Mashimo et al. conducted a descriptive epidemiological study to analyze fall-related deaths in Japan from 1975 to 2019 using national vital statistics data. The findings obtained revealed that fall-related deaths peaked in December, with lower rates being observed in summer [27]. A systematic review by Byrne et al. of 12 studies from nine countries (the United States, Australia, the United Kingdom, Canada, South Korea, Hong Kong, Norway, Spain, and Japan) on the relationship between temperature and falls showed that falls were more common in cold periods, particularly winter, in nine of the studies [28]. 

In the present study, the number of fall-related deaths was the highest in December and was associated with air temperature parameters in a simple correlation analysis. These relationships were weakened after adjustments for age. The effect of the aging rate on the number of fall-related deaths was stronger than that of the air temperature parameter. Since fall-related deaths are more common in winter, it may be necessary to raise awareness about fall prevention in clinical practice. There are some studies on fall prevention in winter [29-31]. In the UK, the charity Age UK runs a winter campaign called 'Spread the Warmth,' which combines cold weather support with information on safe footwear and home safety to help older adults prevent falls [29]. In Canada, November is designated as 'Falls Prevention Month,' and local governments and healthcare providers work together to promote safe walking on snow and ice, the use of slip-resistant footwear, and home fall risk checklists for older adults [30]. In Japan, Sapporo City conducts winter pedestrian fall-prevention campaigns through public-private partnerships, offering guidance on safe walking on snowy roads, fall-prevention classes, pamphlets, videos, and websites to encourage safe winter walking for older individuals and the general public [31].

There are a number of limitations that need to be addressed. This was an ecological study, and we were unable to obtain individual data or the cause of death. Therefore, causal inferences cannot be drawn, and there is a risk of ecological fallacy. Furthermore, we used monthly data from 2017 to 2023. The classification of a fall-related death was changed in 2017 in Japan. Therefore, due to the inability to use data over a long period and the need to rely on a relatively short time series, there may be issues with the accuracy of predictions. In addition, we did not conduct formal out-of-sample validation of the Prophet model and could not adjust for potential confounders such as comorbidities or environmental hazards. Nevertheless, the present results provide useful data for the development of strategies to prevent fall-related deaths.

## Conclusions

This study conducted a detailed analysis of fall-related deaths across Japan, identifying key findings. The Prophet time series analysis projected sustained increases through 2027, with preserved seasonality. These results highlight the necessity of seasonality-informed fall-prevention strategies in Japan, particularly winter-focused interventions. The Prophet projections underscore the urgency of targeted prevention measures. As these forecasts are based on an observational ecological study without formal validation, they should be interpreted with caution. Future research elucidating specific fall etiologies would facilitate more precise interventions.
